# Development of Stable Homozygous Wheat/*Amblyopyrum muticum* (*Aegilops mutica*) Introgression Lines and Their Cytogenetic and Molecular Characterization

**DOI:** 10.3389/fpls.2019.00034

**Published:** 2019-01-31

**Authors:** Julie King, Claire Newell, Surbhi Grewal, Stella Hubbart-Edwards, Cai-yun Yang, Duncan Scholefield, Stephen Ashling, Alex Stride, Ian P. King

**Affiliations:** ^1^School of Biosciences, The University of Nottingham, Loughborough, United Kingdom; ^2^Limagrain UK Limited, Bury St Edmunds, United Kingdom

**Keywords:** wheat, introgression, *Amblyopyrum muticum*, doubled haploid, SNP markers, genomic *in situ* hybridization

## Abstract

Wheat is one of the world’s most important sources of food. However, due to its evolution its genetic base has narrowed, which is severely limiting the ability of breeders to develop new higher yielding varieties that can adapt to the changing environment. In contrast to wheat, its wild relatives provide a vast reservoir of genetic variability for most, if not all, agronomically important traits. Genetic variation has previously been transferred to wheat from one of its wild relatives, *Ambylopyrum muticum* (previously known as *Aegilops mutica*). However, before the genetic variation available in this species can be assessed and exploited in breeding and for research, the transmission of the chromosome segments introgressed into wheat must first be stabilized. In this paper we describe the generation of 66 stably inherited homozygous wheat/*Am. muticum* introgression lines using a doubled haploid procedure. The characterisation and stability of each of these lines was determined via genomic *in situ* hybridization and SNP analysis. While most of the doubled haploid lines were found to carry only single introgressions, six lines carried two. Three lines carried only complete *Am. muticum* chromosomes, 43 carried only small or very small introgressions and the remainder carried either only large introgressions or a large plus a small introgression. The strategy that we are employing for the distribution and exploitation of the genetic variation from *Am. muticum* and a range of other species is discussed.

## Introduction

Wheat is one of the world’s leading sources of food providing circa 20% of the world’s daily intake (Reynolds et al., 2012). Following a history of continued yield improvements by breeders, wheat yields are now plateauing at a time when the world’s population is rapidly increasing (Charmet, 2011). The reason for this plateauing is a lack of genetic variation within modern day wheat varieties compounded by environmental change, i.e., hexaploid wheat only evolved once or twice circa 10,000 years ago and thus it has been through a significant genetic bottle-neck. In contrast to wheat its wild relatives provide a vast reservoir of genetic variation for potentially most, if not all, traits of agronomic importance. In the past there have been several examples of the exploitation of genetic variation from wild relatives for wheat improvement. For example, the transfer of a segment of *Aegilops umbellulata* to wheat conferring resistance to leaf rust (Sears, 1955), the transfer of a segment of *Aegilops ventricosa* carrying resistance to eyespot (Doussinault et al., 1983) and its subsequent release as the variety Rendevouz.

Even though there have been a number of successes in the past, the genetic variation available within the wild relatives remains largely untapped with regard to its exploitation in breeding programs. The main reason for this has been the lack of high throughput technological screens to identify when genetic variation has been introgressed into wheat. A direct result is that where in the 1970s and 1980s there were many hundreds of scientists working in the field there are now very few. However, the advances in technology, e.g., gene and genome sequencing, comparative mapping, molecular marker development etc., over the last 10–15 years has now resulted in the development of systems that can be utilized for the high throughput detection and high-resolution characterisation of wheat/wild relative introgressions. King et al. (2017, 2018) and Iefimenko et al. (2015) used an Axiom array in combination with a specific crossing strategy to generate and identify introgressions from *Ambylopyrum muticum*, *Aegilops speltoides* and *Thinopyrum bessarabicum*. Many hundreds of new introgressions were generated and detected in these works. The frequency of introgression between wheat and *Am. muticum* and *Ae. speltoides* was high enough to generate linkage maps of these species, with over 500 new introgressions developed from *Am. muticum* and *Ae. speltoides* (King et al., 2017, 2018).

In the past much of the work undertaken had been aimed at transferring genetic variation from a wild relative to wheat for a single trait. This strategy normally required the production of an interspecific hybrid followed by the generation of wheat/wild relative addition and substitution lines (King et al., 2016). A chromosome manipulation program was then undertaken to introgress a small chromosome segment, from the chromosome of the wild relative (that carried the gene(s) controlling the target trait) into wheat. The work undertaken by King et al. (2017, 2018) and Iefimenko et al. (2015) used a different strategy. Although *Am. muticum Ae. speltoides* and *Th. bessarabicum* all carry genetic variation for a range of traits such as disease resistance, salt tolerance, etc., the main aim of these works was to introgress the entire genome of these species into wheat in small chromosome segments, i.e., transfer all of the genetic variation in these species into wheat. In the future each of the introgression lines carrying a chromosome segment from these three wild relatives will be screened phenotypically for a range of traits. This strategy will allow the phenotypic analysis of the entire genomes of each of the wild relatives for a wide range of traits (the limiting factor being the number of traits screened for) rather than a single trait.

In order for each of the introgression lines to be analyzed phenotypically they need to be multiplied and stably inherited. All of the introgressions initially produced byKing et al. (2017, 2018) and Iefimenko et al. (2015) are in the heterozygous state with the result that the progeny produced from plants carrying them will segregate for lines with and without the introgression. In contrast, lines homozygous for introgressions are expected to be stably inherited and thus can be multiplied and distributed for large scale trait analysis.

In this work, however, we focused on the development of homozygous *Am. muticum* introgression lines a species that has been shown with limited previous trait analysis to contain genetic variation for environmental stresses (Iefimenko et al., 2015) and powdery mildew (Eser, 1998) and their characterisation via SNP analysis and genomic *in situ* hybridization (GISH). The strategy for exploitation of introgressions is discussed, i.e., all stable homozygous introgressions that are generated will be subjected to a wide range of trait analyses via our collaborators both in the United Kingdom and globally, in order to determine the agronomic and scientifically important genetic variation carried by the *Am. muticum* introgressions.

## Materials and Methods

### Plant Material

**FIGURE 1:**
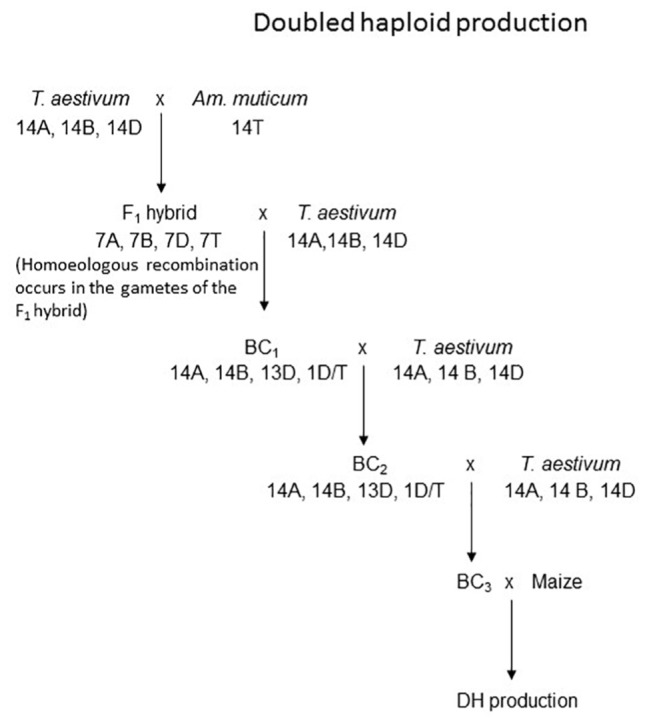
Derivation of the material used to develop DH homozygous introgression lines. This example shows an ideogram of an *Am. muticum*/*T. aestivum* D genome recombinant and the subsequent development of a DH line from it.

Wheat/*Am. muticum* introgressions were generated as described by King et al. (2017). In summary *T. aestivum*, vars. Chinese Spring and Pavon 76, were pollinated with *Am. muticum* (which carries suppressors of *Ph1*/promoters of homoeologous recombination). Accessions 2130004 and 2130012 of *Am. muticum* were obtained from the Germplasm Resource Unit at the JIC, United Kingdom. The F_1_ interspecific hybrids produced were then backcrossed to *T. aestivum* vars. Paragon or Pavon 76 to recover introgressions in a wheat background. The BC_1_ population and its subsequent progeny were also backcrossed to Paragon to produce a BC_3_ populations which were themselves crossed to maize to initiate doubled haploid (DH) production (Figure 1). When the work described in this paper was initially undertaken the Axiom^®^ Wheat-Relative Genotyping Array described by King et al., 2017 was not available. Thus, selection of BC_3_ plants for DH production was based upon the identification of plants carrying introgressions in the BC_2_ individuals via GISH analysis. However, leaf material was taken from each of the BC_3_ plants used for DH production for SNP analysis when the genotyping array became available.

### Doubled Haploid Production

The DH production procedure used was as described by Laurie and Reymondie (1991). In summary 1 day after pollination with maize (cultivars Northern Extra Sweet, Prelude and Sundance), internodes below pollinated spikes were filled with 10 mg l^-1^ of 2,4-dichlorophenoxyacetic acid (2,4-D solution) with a syringe and the holes sealed with petroleum jelly. The 2,4-D solution was also injected into each floret. After 14–21 days embryos were excised and cultured. Colchicine treatment was carried out as described in Nemeth et al. (2015).

### Detection of Wheat/*Am. muticum* Introgressions

#### Marker Analysis

A 35K Axiom^®^ Wheat-Relative Genotyping Array (Affymetrix, Santa Clara, CA, United States) was used to genotype a set of BC_1,_ BC_2_ and BC_3_ wheat/*Am. muticum* introgression lines by King et al. (2017). The genetic map generated for *Am. muticum* by King et al. (2017) was used in conjunction with the 35K Axiom^®^ Wheat-Relative Genotyping Array to detect and characterize *Am. muticum* segments in the DH lines and in the BC_3_ lines they originated from as described in King et al. (2017). [All the SNPs incorporated in the array formed part of the Axiom^®^ 820K SNP array (Winfield et al., 2016) with the dataset for the Axiom^®^ 820K SNP Array available at www.cerealsdb.uk.net (Wilkinson et al., 2012, 2016)]. The SNPs used were polymorphic between *Am. muticum* and the three wheat cultivars used in the generation of the DH lines (Chinese Spring, Paragon and Pavon 76). Also, most SNPs were not genome-specific in wheat, i.e., they had copies on more than one genome of wheat and thus, were unable to distinguish between a heterozygous and a homozygous segment since presence of either type of segment produced a heterozygous call.

#### Cytogenetic Analysis

The protocol for genomic *in situ* hybridization (GISH) was as described in Zhang et al. (2013); Kato et al. (2004), and King et al. (2017). Genomic DNAs was isolated from *Am. muticum* and the three putative diploid progenitors of bread wheat, i.e., *T. urartu* (A genome), *Ae. speltoides* (B genome) and *Ae. tauschii* (D genome). Genomic DNAs of *Am. muticum*, *T. urartu* and *Ae tauschii* were labeled by nick translation with ChromaTide Alexa Fluor 546-14-dUTP, ChromaTide Alexa Fluor 488-5-dUTP [Thermo Fisher Scientific (Invitrogen), Waltham, MA, United States] and Alexa Fluor 594-5-dUTP [Thermo Fisher Scientific (Invitrogen), Waltham, MA, United States], respectively. Genomic DNA of *Ae. speltoides* was fragmented to 300–500 bp at 100°C.

Preparation of chromosome spreads was as described in Kato et al. (2004) and King et al. (2017). Slides were probed using labeled genomic DNAs of *Am. muticum* (100 ng), *T. urartu* (100 ng), *Ae. tauschii* (200 ng) and fragmented genomic DNA of *Ae. speltoides* (5000 ng) as blocker in a ratio of 1:1:2:50 per slide to detect the *Am. muticum* introgressions and the AABBDD genomes of wheat. Slides were counterstained with Vectashield mounting medium with 4′-6-diamidino-2-phenylindole,dihydrochloride (DAPI) and analyzed using a Zeiss Axio ImagerZ2 upright epifluorescence microscope (Carl Zeiss Ltd, Oberkochen, Germany) with filters for DAPI (Ex/Em 358/461 nm, blue), Alexa Fluor 488 (Ex/Em 490/520 nm, green), Alexa Fluor 594 (Ex/Em 590/615 nm, red) and Alexa Fluor 546 (Ex/Em 555/570 nm, yellow). Photographs were taken using a MetaSystems Coolcube 1 m CCD camera. Further slide analysis was carried out using Meta Systems ISIS and Metafer software (Metasystems GmbH, Altlussheim, Germany).

## Results

Sixty-nine BC_3_ plants derived from BC_2_ lines (characterized by GISH and identified as carrying *Am. muticum* chromosomes and introgressions) were pollinated with maize in order to generate DH lines. Subsequent SNP analysis of the 69 BC_3_ plants using the newly developed Axiom^®^ Wheat-Relative Genotyping Array indicated that 57 of the 69 BC_3_ individuals selected carried *Am. muticum* chromosomes and/or wheat/*Am. muticum* introgressions. Of the 12 BC_3_ plants that did not carry *Am. muticum* chromosomes and wheat/*Am. muticum* introgressions 11 (92%) produced DHs (Supplementary Table S1).

**FIGURE 2:**
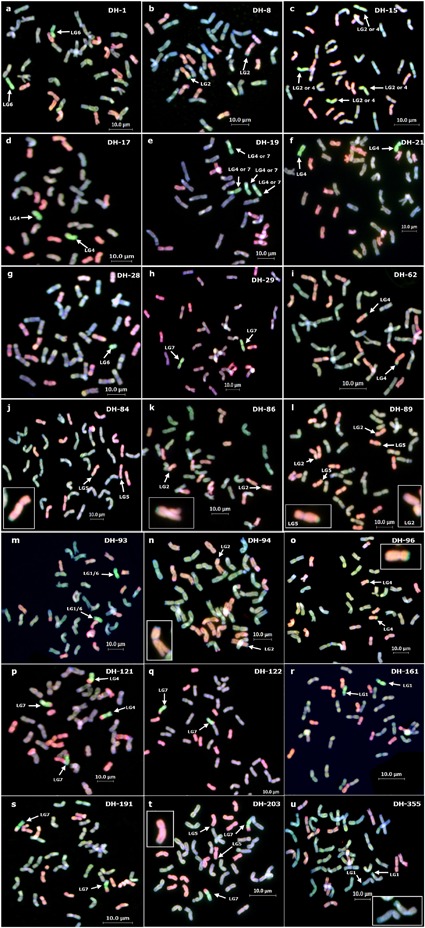
GISH analysis of DH lines showing the different segments present. **(a)** DH-1 **(b)** DH-8 **(c)** DH-15 **(d)** DH-17 **(e)** DH-19 **(f)** DH-21 **(g)** DH-28 **(h)** DH-29 **(i)** DH-62 **(j)** DH-84 **(k)** DH-86 **(l)** DH-89 **(m)** DH-93 **(n)** DH-94 **(o)** DH-96 **(p)** DH-121 **(q)** DH-122 **(r)** DH-161 **(s)** DH-191 **(t)** DH-203 **(u)** DH-355. All GISH was carried out using four colors as indicated in Materials and Methods, but all photos shown were taken using three colors with a green filter for Alexa Fluor 546 for best visualization of the *Am. muticum* segments (bright green segment indicated with white arrows). The A and B genome chromosomes are colored the same (blue/light green) under this color capture. The D genome is shown in red. Small segments are also shown as enlargements.

Of the 57 BC_3_ plants carrying *Am. muticum* chromosomes and/or wheat/*Am. muticum* introgressions 32 (56%) produced DHs. In total 220 DH plants were produced of which 161 (73%) grew and produced seed. The remaining 59 (27%) DHs either died or were sterile. SNP analysis indicated that of the 161 DH plants that set seed, 93 (58%) did not carry any *Am. muticum* chromosomes and/or wheat/*Am. muticum* introgressions (Supplementary Table S1). SNP analysis revealed that the remaining 68 DH plants that set seed all carried one or two wheat/*Am. muticum* introgressions or chromosomes (Table 1 and Supplementary Table S1). Table 1 gives the genome information for one DH plant for each different segment - each of these selected plants is also shown with GISH in Figure 2. Full genome information for all BC3 plants used and all DH plants produced is given in Supplementary Table S1. DH-4 was subsequently lost due to very low seed set and germination. Fifty seven of the lines were analyzed using multi-color GISH (mcGISH; Figure 2 shows the GISH for one DH plant for each different segment) and the linkage group of the *Am. muticum* and/or wheat/*Am. muticum* introgression that each DH derived line was determined via SNP analysis (Table 1). SNP analysis revealed that one of the lines, DH-93, carried linkage group 1L and linkage group 6S markers. However, cytogenetic analysis indicated the presence of a single pair of chromosomes. Since previous work has shown that *Am. muticum* linkage group 6 and group 1 chromosomes are not translocated relative to wheat (King et al., 2017) this observation indicates the presence of a translocated *Am. muticum* 6S.1L chromosome potentially derived from mis-division of complete chromosomes followed by centric fusion.

**Table 1:** Genome information for BC_3_ and DH plants showing the number, linkage groups and size of *Am. muticum* segments present, the wheat genome involved in the recombination (where known) and the number of A, B, and D genome chromosomes.

BC_3_ code	No. of segments in BC3	No. of DH plants produced with segment(s)	DH plant with GISH validation	No. of segments in DH plants	Linkage group of DH segments	Segment size	Wheat genome recombined with	No. of A chromosomes	No. of B chromosomes	No. of D chromosomes	Total No. of chromosomes
165D	2	1	1	1	6	Whole		14/16	14	12	42/44
174D	1	7	8	1	2	Very small	D	14	14	14	42
177A	2	4	15	2	2, 4	Large, Large	B, B	12	14	14	42
			17	1	4	Large	B	14	12	14	42
177B	2	3	19	2	4, 7	Large, Large	B, B	14	12	14	42
			21	1	4	Large	B	14	12	14	42
182B	2	2	28	1	6	Telo		14/15	13/14	14	41/42/43 + /-T
			29	1	7	Whole		14	14	11	41
185A	1	11	62	1	4	Small	D	14	14	13/14	41/42
186A	4	7	84	1	5	Very small	D	14	14	14	42
			86	1	2	Very small	D	12	14	16	42
			89	2	2, 5	Very small, Very small	D, D	12	14	16	42
187A	4	2	93	1	1/6cf	Whole	Centric fusion	12	14	12	40
			94	1	2	Small	D	12	14/15	16	42/43
187B	3	2	96	1	4	Small	D	14	14	14	42
189B	2	3	121	2	4, 7	Large, Large	D, D	14	14	10	42
			122	1	7	Large	D	14	14	12	42
190A	1	15	355	1	1	Very small	A	16	12	14	42
194A	2	1	161	1	1	Large	B	12	14	12	40
197B	1	8	191	1	7	Large	D	14	14	12	42
197E	2	1	203	1	7	Very small, Large	D, D	14	14	12	42


*Of the 68 DH lines with segment(s) only GISH validated DH lines (represented in Figure 2) have detailed information shown above. For complete genome information on all DH lines produced, including those with no segments, see supplemental data (Supplementary Table S1).*

McGISH analysis of the progeny derived from the 67 fertile DH individuals indicated that the wheat/*Am. muticum* introgressions and complete chromosomes were stably transmitted to the next generation with one exception. DH-28 was found to be heterozygous for a telosome derived from *Am. muticum* linkage group 6 (Figure 2g). As a result, the progeny derived from this DH segregated for the presence or absence of this chromosome.

**FIGURE 3:**
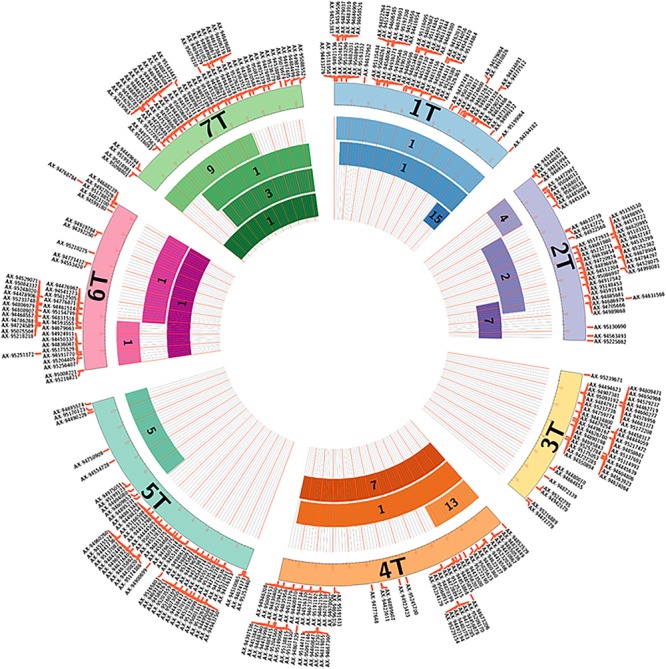
Size of the *Am. muticum* segments within the DH lines and their coverage of the *Am. muticum* genome visualized using Circos v. 0.67 (Krzywinski et al., 2009). The numbers within each segment shows the number of lines containing that segment. The sizes of the chromosomes and the markers on them are obtained from the genetic map of *Am. muticum* (King et al., 2017).

The DH plants produced carried segments from linkage groups 1, 2, 4, 5, 6, and 7 (Figure 3) although the introgressed segments did not cover the whole of these linkage groups. Only one DH plant (DH-4) was found to contain a segment from linkage group 3. However, this plant was subsequently lost as the pollen fertility was very low and thus the line produced very few seed which were shriveled and failed to germinate.

McGISH also revealed that while the introgressions/ chromosomes were largely stably inherited, the number of chromosomes of each wheat genome varied in some of the DH lines (Table 1, Supplementary Table S1 and Figure 2). For example, DH-1 carried a pair of *Am. muticum* group 6 chromosomes but the number of A genome chromosomes varied, i.e., two plants carried 14 A genome chromosomes, 14 B chromosomes and 12 D chromosomes, while a third plant carried 16 A genome chromosomes, 14 B genome chromosomes and 12 D genome chromosomes. In addition to wheat/*Am. muticum* introgressions, several lines also carried intergenomic wheat recombinants, e.g., A/B, A/D recombinants.

## Discussion

In the past, attempts to introduce genetic variation from a wild relative have generally focused on introgressing a single chromosome segment carrying genetic variation for a single trait [frequently using substitution lines/addition lines as a starting point (King et al., 2016)]. In contrast the objective of the work described here is not focused on just single traits, i.e., we are attempting to identify useful genetic variation for a wide range of traits from *Am. muticum* for future exploitation. In order to do this, we aim to generate very large numbers of introgressions in wheat from *Am. muticum* (ideally, we would like to introgress the entire genome of *Am. muticum* into wheat). In order to identify as much genetic variation as possible, a wide range of trait analyses will be performed on each introgression line generated (by ourselves and collaborators in both the public and private sectors globally). In addition, all lines derived from the (BBSRC funded) Wheat Research Centre at the University of Nottingham will be made available upon request (subject to handling charges, e.g., phytosanitary certificates).

A key factor in this strategy is the bulking and distribution of seed for trait analysis. However, before seed can be bulked each individual introgression must first be in a homozygous state to ensure that it is stably inherited to the next generation (all the introgressions we generate are initially in a heterozygous state and thus, any progeny derived from them will segregate for their presence and absence). The generation of DH lines in the work described in this paper represents one of the methods we are employing to generate homozygous introgression lines. In this work, 56% (32) of BC_3_ plants carrying an *Am. muticum* chromosome or introgression produced DHs as compared to 92% (11) of BC_3_ plants which did not carry *Am. muticum* chromosomes or introgressions. From the 32 BC_3_ plants a total of 220 DH plants were produced, but only 68 of those that produced seed carried *Am. muticum* introgressions or chromosomes (with one of these lines being subsequently lost). These results indicate that the DH technique has resulted in the successful generation of homozygous introgressions albeit at a relatively low frequency. However, further work is required to optimize the protocols used to increase the frequency of DH generation from lines carrying introgressions and chromosome segments from the wild relatives of wheat, e.g., 2,4-D concentration, timing of embryo excision, colchicine concentration, etc.

One of the key objectives of the work outlined above is to introgress the entire genome of *Am. muticum* into wheat. The lines described here do not cover the entire genome as shown in Figure 3. In particular, the stable lines produced do not contain any segments from linkage group 3 of *Am. muticum*. However, it is difficult to establish at this stage if the regions not represented point to regions of the genome that are recalcitrant to transmission or are simply not represented due to the relatively small sample size. We also did not observe any examples of where an introgression was detected by SNP analysis that was not detected by GISH analysis (cryptic introgressions). However, again due to the relatively small sample size, it was not possible to determine if cryptic introgressions do or do not occur.

Initially, lines homozygous for large introgressions are being generated, distributed, e.g., Australia, United States, commercial breeding companies, and are being used for preliminary trait analyses. This initial analysis will enable the determination of which regions of the genome of *Am. muticum* carry genetic variation for target traits. The second stage of analysis will focus on the analysis of small introgressions derived from the large regions that have been found to carry target genetic variation (homozygous lines will need to be generated for each of the small introgression lines prior to the distribution for trait analysis). In this way we will identify the smallest introgression that carries the gene(s) controlling the target trait (the smaller the introgression the less likely it will be that it will carry deleterious genes in addition to the target gene). If small introgressions are not available, then overlapping introgressions will be intercrossed to produce smaller ones as described by Sears (1955) or further introgressions will be generated.

A further requirement of the strategy being undertaken is that once homozygous, each introgression must be stably inherited. Out of the viable 67 DH lines generated only one, DH-28 (1.5%), was not stably inherited. The remaining 66 DH (98.5%) were found to be stably inherited.

A number of abnormalities were observed within the wheat genome, e.g., the number of chromosomes of the three wheat genomes was occasionally found to vary from the euploid condition (i.e., 14 A, 14 B and 14D chromosomes). In addition, intergenomic recombinants were observed between the three genomes of wheat. The reason for these abnormalities may result from the strategy that was employed to generate introgressions, i.e., euploid wheat was pollinated with *Am. muticum* to produce an interspecific F_1_ hybrid which was then backcrossed to euploid wheat to produce a BC_1_ population. The F_1_ hybrids generated were haploid for each of the three wheat genomes and the *Am. muticum* genome and thus the only recombination that could occur was between homoeologous chromosomes (King et al., 2017, 2018). However, while this strategy resulted in the generation of a high frequency of wheat/*Am. muticum* recombination and hence introgressions it also appears to have led to the generation of homoeologous recombination between the three genomes of wheat.

The variable aneuploid number of A, B and D genome chromosomes was probably also derived from the interspecific F_1_s, i.e., the haploid genome complement of the F_1_ would have resulted in the production of unbalanced gametes and thus variable numbers of A, B and D genome chromosomes in the BC_1_ generation. In order to restore the diploid chromosome complement of the wheat genome and to remove any wheat/wheat intergenomic recombinants, further backcrossing will be required.

Of the 66 stable DHs generated, 23 that carry large segments, and upon request from our collaborators a further five carrying small introgressions, have now been released. The remaining DHs will be made available in the near future. In this program, we have demonstrated that DH procedures can be used to generate homozygous introgression lines. However, in addition to using DH procedures we are also generating homozygous introgression lines via self-fertilization of heterozygous lines and progeny testing. To assist us in identifying homozygous introgression lines (produced either by DH technology or by self-fertilization) we are developing circa 1000 KASP markers to facilitate selection.

In this paper, we have only described work on one wild relative, i.e., *Am. muticum*. However, we are working on a number of other species and we aim to use DH techniques and self-fertilization to initially produce large homozygous introgressions that span the genomes of these relatives (and smaller homozygous introgressions as required). Thus, over the coming years, many hundreds of homozygous introgression lines will be made available for trait analysis. In this way we intend to facilitate the large-scale exploitation of genetic variation from the wild relatives of wheat for wheat improvement.

In the past, there has been some reticence in using genetic variation from the wild relatives of wheat, mainly stemming from the fact that target genes may also be associated with deleterious genes. However, the development of new technologies provides the means by which this problem can now be overcome. We believe the biggest threat to the exploitation of genetic variation from wheat’s wild relatives, lies in the fact that whereas there were hundreds of active scientists in the field in the 1970s and 1980s, very few with the requisite expertise now remain.

## Author Contributions

JK, SG, C-yY, SH-E, DS, SA, and IK carried out the crossing program. CN and AS carried out the doubled haploid production. SG analyzed the genotyping data. C-yY carried out the genomic *in situ* hybridization. IK wrote the manuscript with assistance from JK. All authors have read and approved the final manuscript.

## Conflict of Interest Statement

The authors declare that the research was conducted in the absence of any commercial or financial relationships that could be construed as a potential conflict of interest.
